# Supplementary material for: Space-efficient optical computing with an integrated chip diffractive neural network
Source: Nat Commun. 2022 Feb 24;13:1044. doi: 10.1038/s41467-022-28702-0 (PMC8873412; doi:10.1038/s41467-022-28702-0)
Supplement: Supplementary file 1 — Supplementary Information [file 41467_2022_28702_MOESM1_ESM.pdf]

SUPPLEMENTARY INFORMATION FOR

Space-Efficient Optical Computing and An Integrated Chip of Diffractive Neural Network

Zhu *et al*.

Supplementary Note 1: Superiorities of our integrated chip in footprint, power consumption and scalability.

The physical footprint of the IDNN is dependent on the size of MZI modulators and diffraction regions. Neglecting the electrical control lines for tuning each MZI modulator, the total footprint of the IDNN can be estimated as

$$S = N \cdot S_{MZI} + 2 \cdot S_{DIFF} \tag{S1}$$

where S_{MZI} is the area of a single MZI element, N is the input data dimensions and S_{DIFF} is the area of a diffractive region.

Assuming there is a 10 × 10 network, a conservative MZI length is 100 μm and a diffraction region is around 0.04 mm^2, we estimate that the entire chip would be 0.53 mm^2, while for MZI-based OIU [1-3], the area of the whole chip is calculated to around 5 mm^2. Hence our proposed IDNN chip shows superior area efficiency when scaling the network dimension. Neural networks routinely have hundreds of millions of neurons with a huge weight matrix. It has been established that the original weight matrix can be partition into blocks of square circulant matrices allowing the traditional fully connected weight matrix to be replaced with a sequence of smaller circulant matrices without sacrificing effectiveness of the network [4]. Therefore, our IDNN chip can potentially realize hundreds of millions of neurons optically in a scalable and power-efficient manner.

In addition, in our experiment, the resistance of each heater is about 350 Ω, and the average electrical power required for a 2π phase shift is 0.77 mW (2.2 mA). In the IDNN chip, only the MZI modulators need to be modulated via heaters and the number of employed modulators is *N*. As a result, the power consumption of our IDNN chip is

significantly reduced compared to conventional on-chip ONNs which typically need N^2 MZI modulators [1-3]. In **Fig. S1a**, we depict the footprint and power consumption varying with the mode number in a traditional MZI-based network. It can be seen that the footprint and power consumption will increase quadratically with the input data dimension. In our experiment, we designed a network with 10 modes, that only has a hardware footprint of 0.53 mm^2 and power consumption of 17.5 mW, which is less than the traditional MZI-based design. When it extends to 64 modes, our design only needs 3.2 mm^2 area and 112 mW power consumption, compared with traditional 213 mm^2 area and 122.88 W. This means that our designed integrated photonic circuits can achieve higher densities of optical neurons and pave the way for super-large scale and programmable photonic neural networks.

The level of loss sustained in a photonic matrix implementation limits its scalability. This loss is mainly incurred by three parts: (a) the propagation loss in waveguides; (b) photoelectric conversion loss in photodetectors; (c) an extra electrical loss to maintain the phase of heaters. The propagation loss is attributed to two main parts: waveguide scattering loss and loss arising from the diffraction cell. The tested waveguide propagation loss in our experiment is as low as 1.5 dB cm^{-1} and the multi-mode interferometer (MMI) loss is 0.2 dB. Based on the principle of the overlap integral [5], the power received by the array waveguide can be calculated to be 78% of the input power. The loss of 1 dB of the input power can be attributed to the mode mismatch between the slab and output array waveguides, which can be reduced by introducing mode converters between them. **Fig. S1b** shows the simulated optical loss for matrix sizes up to 64 × 64 using experimental loss data, illustrating our design has a strong scalability.

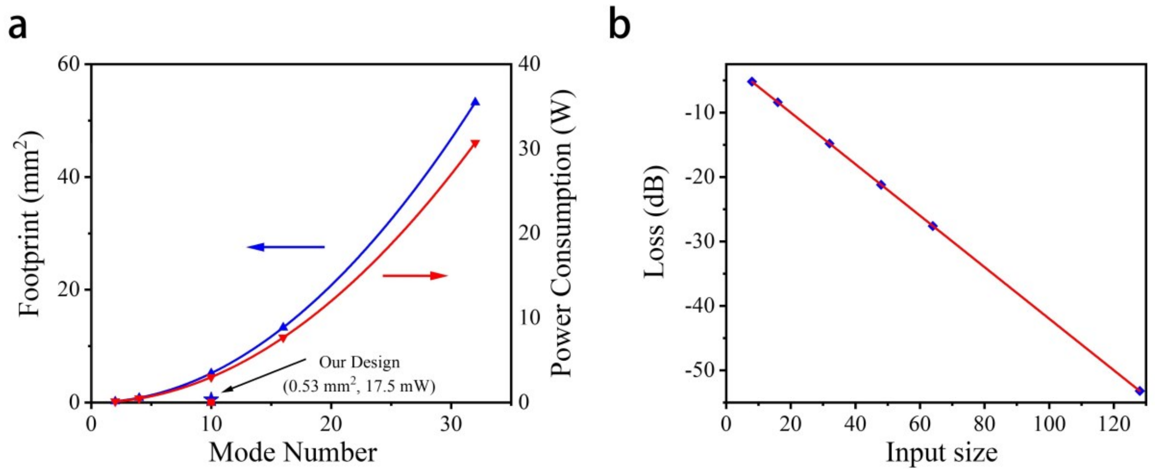

Fig. S1. a. The footprint and power consumption versus mode number increases in traditional MZI-based network and our chip level in two aspects. For our experimental results, our design can achieve 10 × 10 matrix with 0.53 mm^2 area and 17.5 mW energy consumption. **b. Optical loss of the matrix as a function of input data size.** In our experimental results, a directional coupler loss of 0.2 dB and a diffractive cell loss of 1 dB.

Supplementary Note 2: Experiment setup

To measure the output intensity signal, a coherent laser (Santec TSL-510, Santec, U.S.A.) was used as the light source. A polarization controller was used to rotate the polarization of the input light to ensure the coupling of the TE mode light. A thermoelectric controller (TEC) as the substrate under the chip, can be used to control and stabilize the temperature using a temperature controller. Added TEC can further reduce the heat fluctuations caused by ambient temperature and the heat crosstalk within the chip. The circuit which provides the electrical power to phase shifters has a 16-bit output precision. The output light

intensities are acquired by a gainable trans-impedance amplifier and an Analog-to-Digital convertor (NI-9215, U.S.A.) with a resolution of 16 bit.

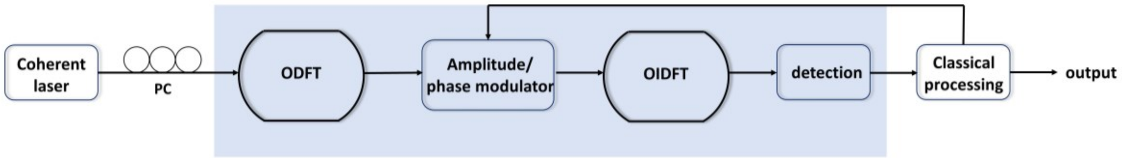

Fig. S2. The workflow of the IDNN system.

Supplementary Note 3: Wave analysis.

The propagation process of light in the slot waveguide area can be realized by the following Fourier transform formula:

$$E_i(k) = \frac{1}{j\sqrt{\lambda_0 R/n_s}} \int E_0(x)\, exp(-j2\pi f_k x)\, dx \tag{S2}$$

where f_k is the angle spectrum of the incident light field and $f_k = kn_s/(\lambda_0 R)$. λ_0 is the light wavelength in vacuum, R is focal length, n_s is the effective refractive index of light propagating in the slot waveguide area, and $E_i(k)$ is normalized electric field after the ODFT operation.

Based on the principle of superposition integration [6], we further calculate the coupling coefficient of each array waveguide with the incident light field imaged on the receiving surface (E_i):

$$\eta_l = \int E_i(k) E_a^*(k + l d_a)\, dk \tag{S3}$$

where η_l is the coupling coefficient of l th array waveguide and E_a is distribution of eigenmodes at the entrance of the array waveguide, and d_a is the array waveguide spacing.

The output field distribution of the array waveguide can be expressed as:

$$E_o(x_2) = \sum_l \eta_l E_a(x_2 + l d_a)\, e^{-j\Delta\phi} \tag{S4}$$

where $\Delta\phi$ is the fixed phase difference of light propagating in the array waveguide, and $E_o(x_2)$ is output field distribution of the array waveguide."

Then, the output field of the array waveguide will go into the second slot waveguide area. The simulation of this area is the same as the simulation of the first one, using the principle of Fourier transform to image the output light field distribution of the array waveguide E_o incident on the receiving surface of the output waveguide:

$$E_{out}(k_2) = \frac{1}{j\sqrt{\lambda_0 R/n_s}} \int E_o(x_2)\, exp(-j2\pi f_{k_2} x_2)\, dx_2 \tag{S5}$$

$$\eta_{out} = \int E_{out}(\zeta_2) E_{a'}^*(k_2)\, dk_2 \tag{S6}$$

where E_0 is the eigenmode of the output waveguide, $E_{a'}$ is a distribution of eigenmodes of the output array waveguide, and $|\eta_{out}|^2$ is the energy received by the output waveguide.

Following the diffraction **Eq. S5**, we can simulate the computing results when the light goes through our IDNN. Assuming the light inputs from the centre waveguide, the simulated electric field distributions from waveguides along the k_1 axis is shown in **Fig. S3c**, which is a Gaussian distribution. These electric field distributions with an added phase difference among the array waveguides propagate the second slab waveguide region, and field distributions along the k_2 axis are depicted in **Fig. S3d**. One notices that the signal is retrieved after going through two diffractive cells (ODFT and OIDFT operations).

We find that the core of the DFT operation is to keep a suitable phase difference of the incident signals from different waveguides before the diffractive cell. As shown in **Figs. S4c and d**, different amplitude distributions are produced if the input signals have different phase differences. The phase difference among the waveguides can be calculated based on

the number of waveguides [6]. The phase shift, ϕ_{n-k}, between input k and output n can be designed to satisfy the following relation by setting positions of input and output waveguides:

$$\phi_{n-k} = \frac{2\pi}{N}\left(k - \frac{N-1}{2}\right)\left(n - \frac{N-1}{2}\right) \tag{S7}$$

We add the following phase offset, φ_k, before the input k by using length adjustment or a phase shift:

$$\varphi_k = \frac{\pi}{N}(N-1)k \tag{S8}$$

Then the phase difference $\triangle\,\phi_n$ between two paths to the output n originating from the inputs $k+1$ and k is derived as

$$\triangle\,\phi_n = (\phi_{n-(k+1)} + \varphi_{k+1}) - (\phi_{n-k} + \varphi_k) = \frac{2\pi n}{N} \tag{S9}$$

Eq. (S9) indicates that the ODFT operation can be realized with the diffractive cell.

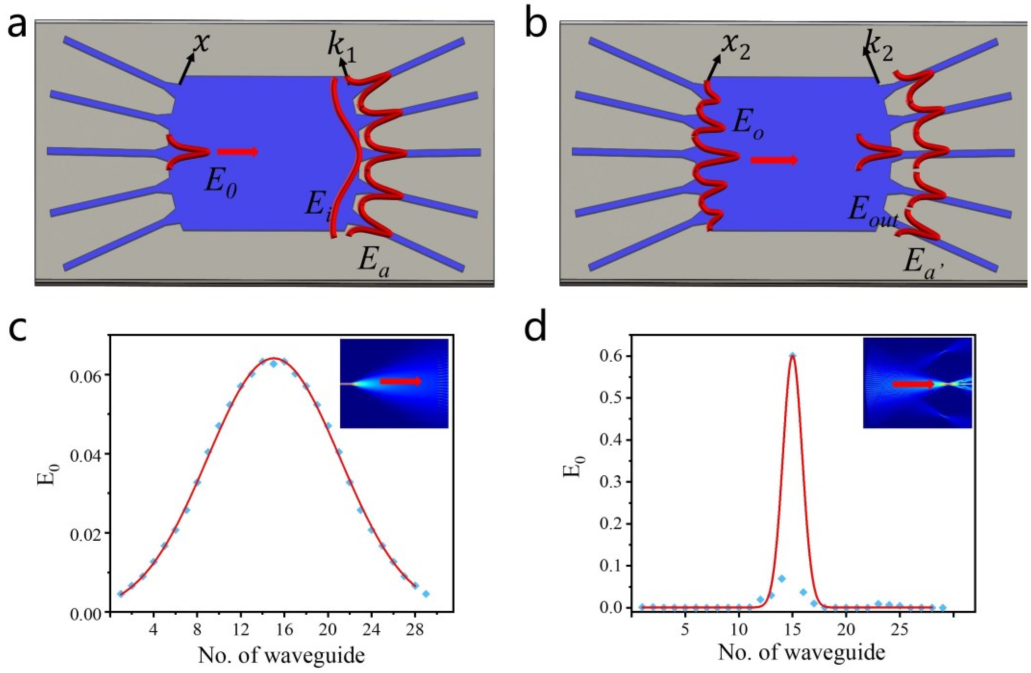

Fig. S3. Wave propagation within the diffractive cells to the ODFT and OIDFT operations. **a** The schematic image of electric field distributions on the diffraction cell when the light inputs from the center waveguide. **b** The schematic image of electric field distributions on the diffraction cell when the result of the ODFT operation E_a is entered as an input signal. **c** The simulated electric field distributions from waveguides among the k_1 axis when signal inputs from the center waveguide. **d** The simulated electric field distributions from waveguides among the k_2 axis when the output results from the ODFT operation are retrieved as the input signal of the OIDFT operation.

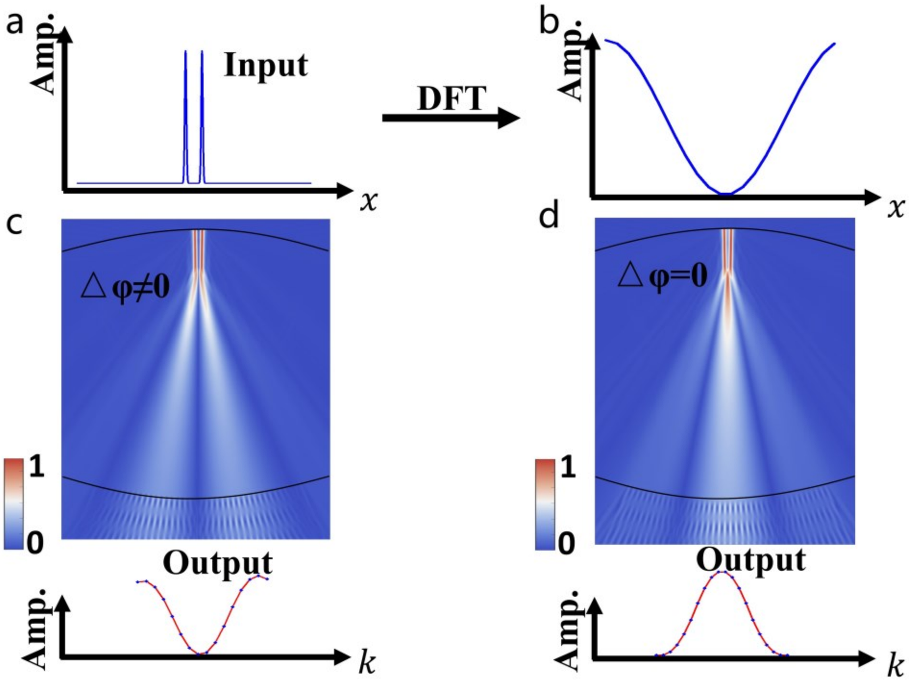

Fig. S4. a The amplitude of input signal. **b** The theoretical DFT results of the input signal. **c** The amplitude information of the wave that is propagating within the diffraction cell with a stable phase difference $\Delta\varphi$ between two paths of input. The output result is the Fourier transform of the input signal. **d** The amplitude information of the wave that is propagating

within the diffraction cell without a phase difference $\Delta\varphi$ between two paths of input. The output result is different from the Fourier transform of the input signal.

Supplementary Note 4: Experimental calibration.

The chip calibration is composed of two parts: the calibration of the inner phase and the outer phase. The transform matrix of the MZI can be determined as

$$U_{MZI} = ie^{i\frac{\theta}{2}} \begin{bmatrix} e^{i\phi} sin\frac{\theta}{2} & e^{i\phi} cos\frac{\theta}{2} \\ cos\frac{\theta}{2} & -sin\frac{\theta}{2} \end{bmatrix} \tag{S10}$$

Where θ is the angle of an inner phase shifter and ϕ is the angle of an outer phase shifter. We first calibrate θ from T1 to T20 (**Fig. S5c**) through the output interference intensity results of each MZI, and the result is shown in **Fig. S6**. Given Eq. (2), if we input light from the upper arm of the MZI and measure the intensity at the lower exit port with a photodiode, the normalized readout voltage would be $V = \left|e^{i\phi}\cos\frac{\theta}{2}\right|^2 = \frac{1+\cos\theta}{2}$. The phase shifter is controlled by a heater and when the power is switched on, the heater induces a refractive index change which causes a relative phase difference between the two arms. Here the calibration of θ is done by increasing the power to the phase shifter, while measuring the optical power output at the corresponding optical port. The power-phase relation can be represented as $\theta(I)= \omega\, I^2+\theta_0$ where I is the current supplied to the heater, ω is a constant related to resistance and the material property and θ_0 is the initial phase difference between the two arms. The final form for the normalized output voltage can be written as $V = V_0 + V_0 k \cos^2(\omega\, I^2 + \theta_0)$. where k is the visibility of the fitting curve and

V_0 is the maximum amplitude of the signal. The measured interference pattern shows the periodical change by the increase of I^2 in **Fig. S6a**.

We calibrate the outer phases ϕ from P1 to P10 by detecting Port0 with the maximum interference intensity. Based on the calculation equation of DFT (**Eq. (S9)**), the signal intensity of Port0 is the sum of light intensities from all input ports. The relative phase of each channel can then be calibrated to achieve the ODFT operation. One notices that the additional phase compensation ($\theta / 2$) should be added to the phase term ϕ if the inner phase shifter change θ, according to the **Eq. (S9)**. Therefore, the initial phase is a baseline, if θ changes, ϕ will compensate accordingly to maintain the accuracy of the calculation. The outer phase ϕ from P11 to P20 also can be calibrated in the same way.

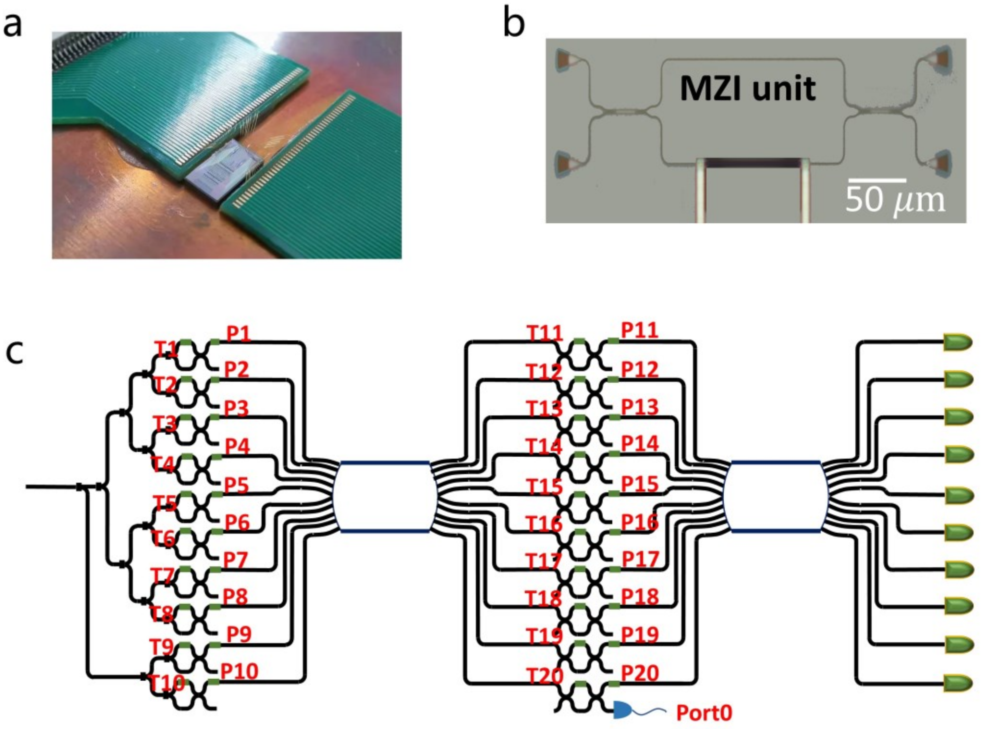

Fig. S5 **a** Image of the chip packaged to a control PCB. **b** The microscope image of the MZI unit. **c** The schematic of the whole chip with heaters.

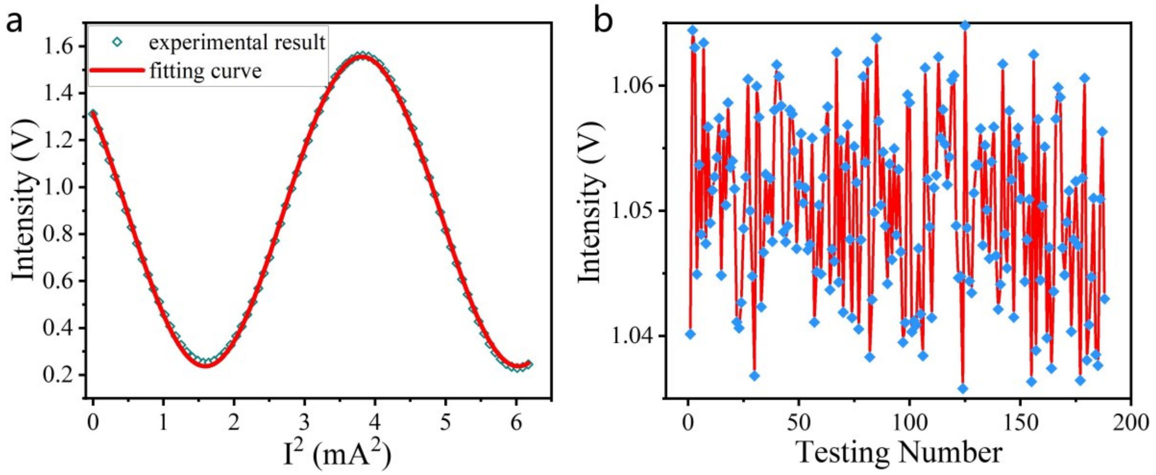

Fig. S6 a The transmission curve for tuning the internal phase shifter. **b** Experimental results of the stability test of the heater.

Supplementary Note 5: Mathematical elucidation of correlation algorithms

In the first task of pattern recognitions of 1D sequences and 2D digit images, the goal is to obtain the similarity between two sequences or images. The cross-correlation, which is a commonly used metric for the similarity between two series, is represented by the symbol $R_{f,g}[n]$ as

$$R_{f,g}[n] = cross - correlation\ (f[j], g[j]) \tag{S11}$$

where $f\ and\ g$ are two series and *n* and *j* are the position numbers of the discrete sequence. The mathematical calculation of the correlation is the same as the convolution, except that the signal is not reversed before the multiplication process. Then, the relation between correlation and convolution is expressed as

$$R_{f,g}[n] = f[n] * g[-n] \tag{S12}$$

where $*$ represents the convolution. The convolution is further converted into the point-wise multiplication in the Fourier transform region, and rewritten as

$$R_{f,g}[n] = \mathrm{IDFT}\{\mathrm{DFT}\{f[n]\} \circ \mathrm{DFT}\{g[-n]\}\} \quad \text{(S13)}$$

where DFT$\{f\}$ and DFT$\{g\}$ are the discrete Fourier transforms of $f[n]$ and $g[-n]$, respectively. $\circ$ is the Hadamard product, which is also known as the element multiplication. By setting two vectors $U = [u_1, u_2, \dots, u_n]$ and $V = [v_1, v_2, \dots, v_n]$, the Hadamard product of the two vectors can be expressed as

$$W = U \circ V = [u_1v_1, u_2v_2, \dots, u_nv_n] \quad \text{(S14)}$$

Finally, the correlation is converted to calculate the Hadamard product between the Fourier transforms of the input series *f* and the target series *g*. Since the Fourier transform converts real numbers to complex numbers, the Hadamard product in our operation is for complex numbers. In our chip, the complex numbers are easily encoded into the amplitude and phase components with the MZI modulators instead of real and imaginary parts. According to the transfer function (**Eq. (S10)**), the output field after the MZI modulator can be expressed as

$$E_{out} = Ae^{i\alpha}E_{in} = ie^{i\left(\phi+\frac{\theta}{2}\right)}\sin\left(\frac{\theta}{2}\right)E_{in} \quad \text{(S15)}$$

where the amplitude component is encoded into θ and phase component is encoded into $\phi + \frac{\theta}{2}$.

As a result, the correlation can be achieved by modulating the MZI using phase and amplitude modulation to evaluate the similarity between two series.

Supplementary Note 6: Image recognition

In the process of sequence recognition, we note that if the corresponding intensity of the entire sequence to be tested is relatively high, strongest light may also be detected at the feature point (maximum intensity position). When the sequence to be tested is longer than the target sequence, the sequence with the overall strong intensity is more likely to appear. To solve this problem, we need to further normalize the detected data by considering both the intensities of the sample and aim sequences.

$$I_{norm}(n) = \frac{I(n)}{I_{sample}} \bullet \frac{I_{aim}}{I_0} \quad \text{(S15)}$$

Where, I_{sample} can be obtained by setting the MZI on the modulation region as a bar state and measuring the intensity sum of the sample sequences at the detectors. I_0 is the intensity of the feature point of $I(n)$, and I_{aim} is the light intensity sum when the signals of the target sequence are received by the detectors.

The experimentally retrieved sequences for different input sequences are shown in **Fig. S7** and 1D sequence recognition with correlation algorithm is experimentally show in **Fig. S8**.

For the 2D image recognition, we use 5×5 binarized matrices to express digits. The correlation results for digits (1, 4, 9) are shown in **Fig. S9**. Because the digits of *4* and *9* are highly similar, the correlation result has a large peak in center of the matrix.

We further discuss the issue of applying the Fourier-based convolution in CNN. For the classical electronic network, the implementation of convolution in Fourier-space has no distinct acceleration advantage for small kernel size that is often used in CNN. As we know, the Fourier-based convolution has three main computing parts: Fourier transform, element multiplication, and inverse Fourier transform. In the optical field, as Fourier transform and inverse Fourier transform can be implemented passively without resource consumption, Fourier-based convolution can still realize a computational acceleration for the small kernel size used in CNN.

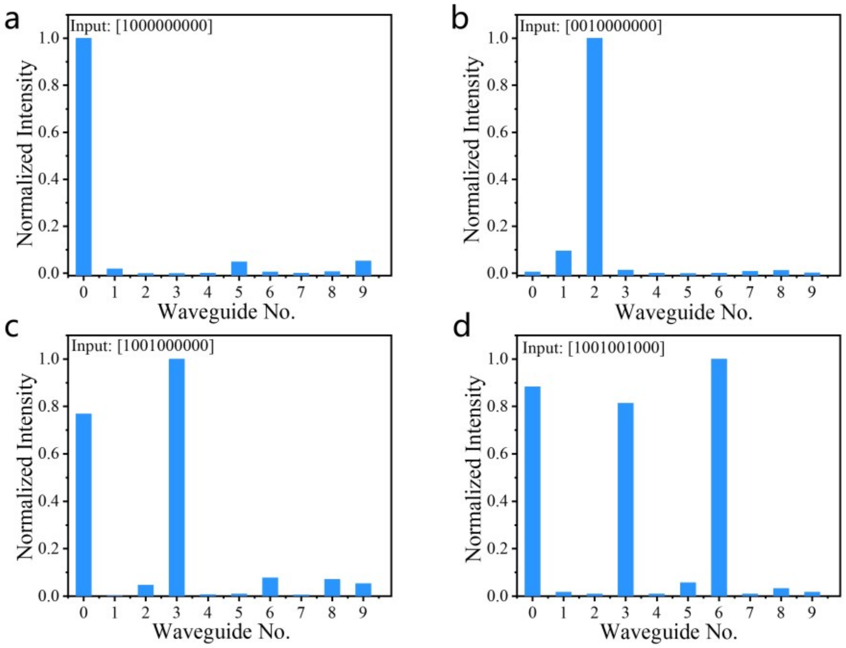

Fig. S7. The experimental retrieved sequence with different input sequences. a [1000000000]. **b** [0010000000]. **c** [1001000000]. **d** [1001001000].

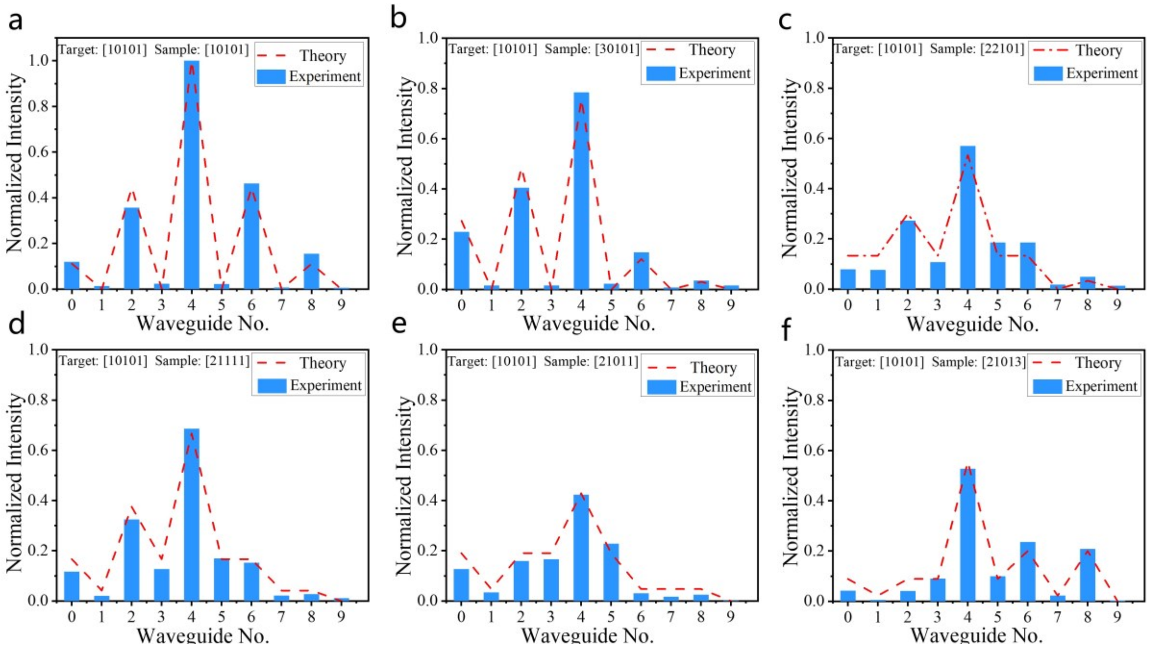

Fig. S8. The experimental results of correlation calculation. The red dashed line is the theoretical result and the histogram is the normalized intensity from nine waveguides.

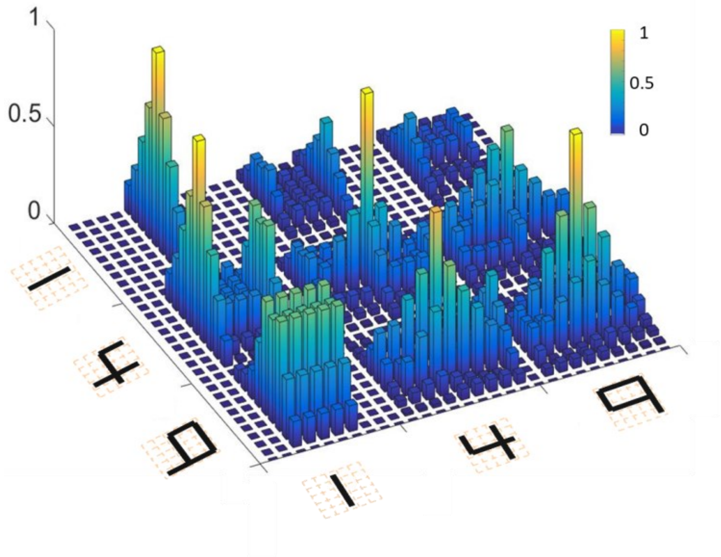

Fig. S9. Experimental correlation results for digit image recognition. There are three digit images (1, 4, 9), and each digit image is composed of *5 × 5* matrix.

Supplementary Note 7: Iris flower

Combined with **Eq. S10**, for amplitude modulation, the additional phase compensation ($\theta/2$) should be added to the phase term ϕ. However, there is only one parameter ϕ that needs to be modulated with phase-only modulation. It is also meaningful if we can only utilize the phase channel of the modulators to achieve the classification. Because it is easier to achieve in an experiment considering only half heaters are needed to add voltage to modulate the phase.

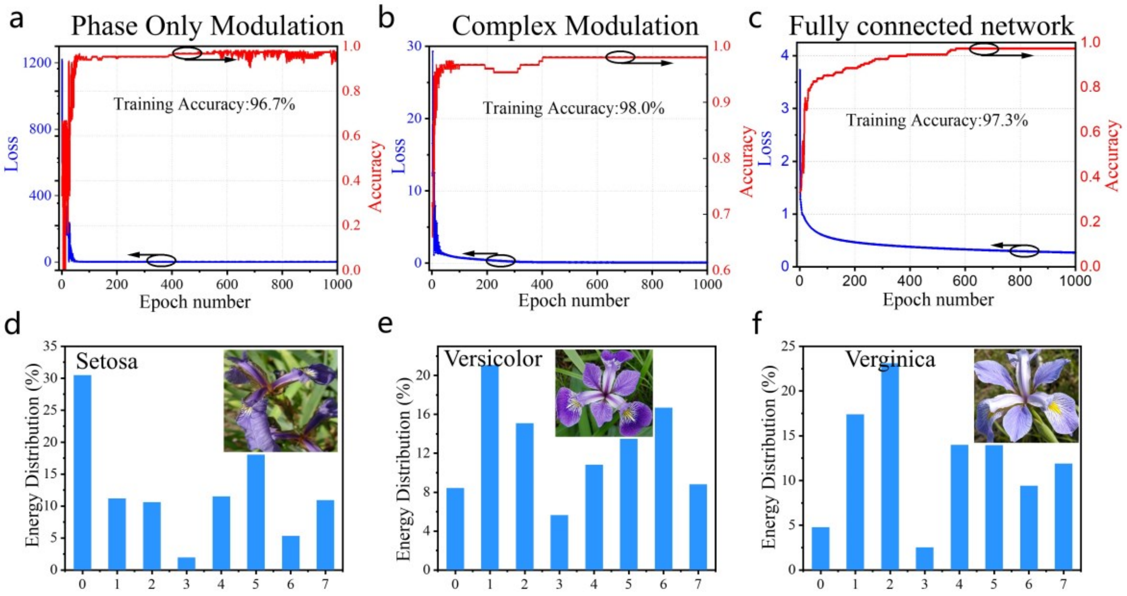

Fig. S10 a, b, c The red and blue lines represent the accuracy and the cost of training with phase-only, complex-valued modulation as well as the fully-connected network. **d, e, f** The experimental results of the output intensity distributions of three different class flowers are demonstrated.

Supplementary Note 8: *MNIST* dataset

The *MNIST* dataset, which includes 10 classes (from 0 to 9), has 60000 images for training and 10000 images for testing. The image data is first compressed to 16 inputs by converting the 28 × 28 grayscale images to the *k*-space and extracting the low-frequency information (4 × 4 matrices in the center of the image). The input layer is a fully connected layer from 16 complex inputs to 10 hidden neurons, the hidden layer consists of a 10 × 10 complex circulant matrix achieved by our chip and the intensity detection outputs are the classification results. The input layer is calculated by the traditional computer and the

output results of the input layer are converted amplitude or phase information as the input of the chip. For amplitude encoding, the amplitude part of the complex output of the input layer will be selected as the input of our chip. For phase encoding, the phase part of the complex output of the input layer will be selected as the input of our chip. The network is implemented using Python (version 3.5.0.) and Pytorch framework version 1.4.0 (Google Inc.) in a desktop computer (Intel ® Xeon ®CPU E5-2650 CPU @ 2.20 GHz, Nvidia GeForce RTX 2080 GPU, 128 GB RAM, Ubuntu 18.04, running a Windows 10 operating system, Microsoft).

In Fig. S11, we simulate the blind testing accuracy results under the phase-only modulation and complex modulation with one-layer and three-layers IDNN network. In these results reported in **Fig. S11**, a single layer IDNN network can achieve 91.8%- and 92.5%-blind testing accuracy for phase-only and complex modulation, respectively. Whereas the numerical results show an accuracy of 92.7% and 93.5%, respectively for phase-only and complex modulation when the hidden layer increases to three layers.

Fig. S11c further demonstrates the confusion matrix for our numerical testing results for 10,000 different handwritten digits. We select ten different handwritten digit images among the testing image set for which numerical testing was successful and simulate their energy distribution, as shown in **Fig. S11f**. A summary of some of the experimental results achieved with our handwritten digit classification IDNN is depicted in **Fig. S12**.

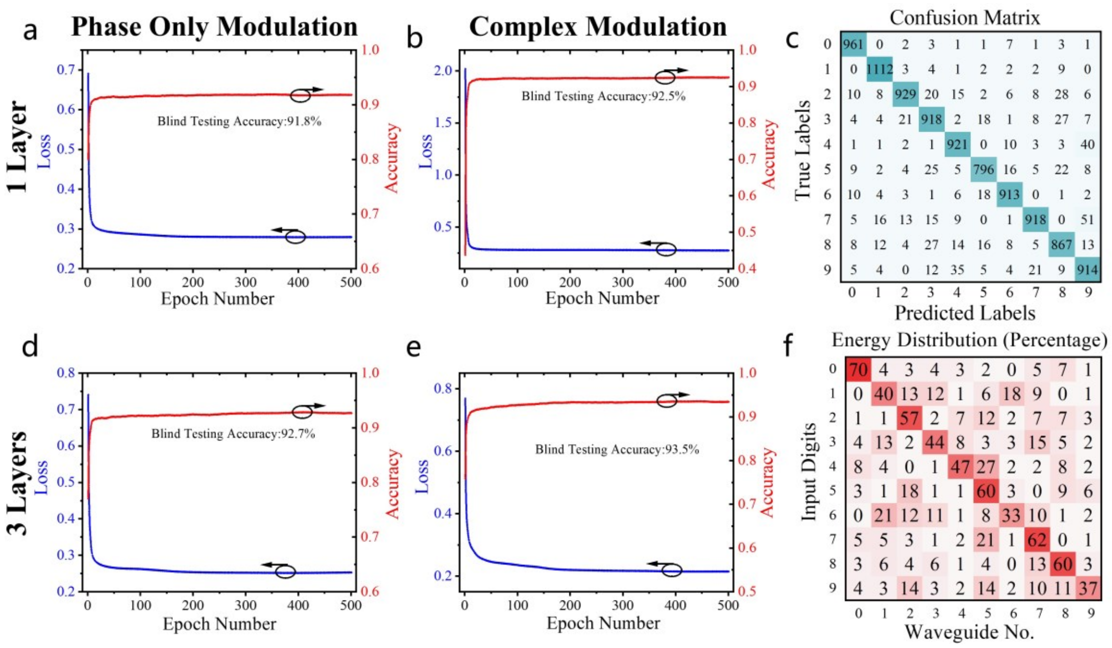

Fig. S11. MNIST results achieved with the IDNN framework. a. b. d. e. Training convergence plots of phase-only as well as complex-valued modulation IDNNs (for 1 layer and 3 layers). The y-axis values in each plot report the MNIST classification accuracy and the loss values as a function of the epoch number for the testing datasets. **c. f.** Confusion matrix and energy distribution percentage for our numerical testing results for 10,000 different handwritten digits (~1000 for each digit), achieving a classification accuracy of 92.5%.

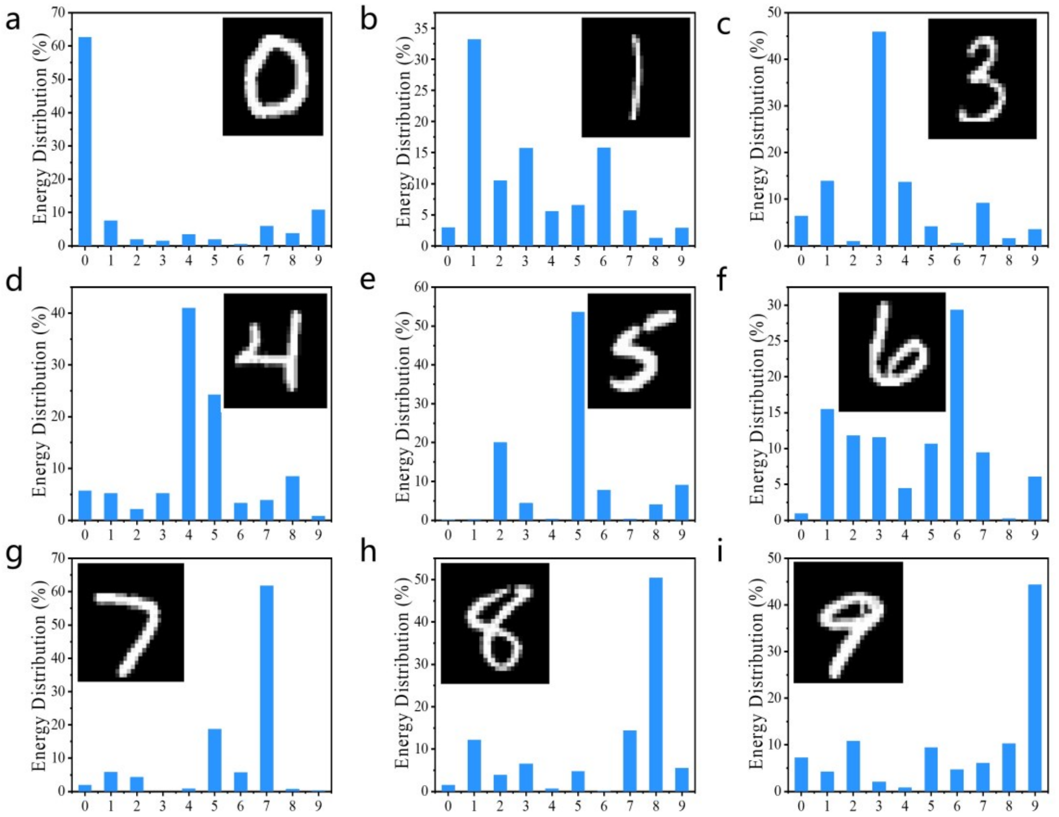

Fig. S12. Sample experimental results for digit classifier IDNN. Summary of some of the experimental results achieved with our handwritten digit classification IDNN. The energy distribution percentage corresponding to each digit at the output plane shows that IDNN has the maximum energy focused on the target detector region of each digit (also see Fig. 5 of the main text).

Supplementary Note 9: *Fashion-MNIST* dataset

In this part, we test the classification performance of the IDNN framework with a more complicated image dataset- the Fashion MNIST dataset, which includes 10 classes with each representing a fashion product (t-shirts, trousers, pullovers, dresses, coats, sandals, shirts, sneakers, bags, and ankle boots). The classification accuracy and the loss values as a function of the epoch number for the testing datasets are shown in **Fig. S13** and the testing accuracies

of the IDNN with a phase-only and a complex modulation are 80.3% and 81.7%, respectively. The complex modulation accuracies are higher than that of phase-only modulation both with one and three hidden layers. The summary of some of the experimental results achieved with fashion product classifications is depicted in **Fig. S14**.

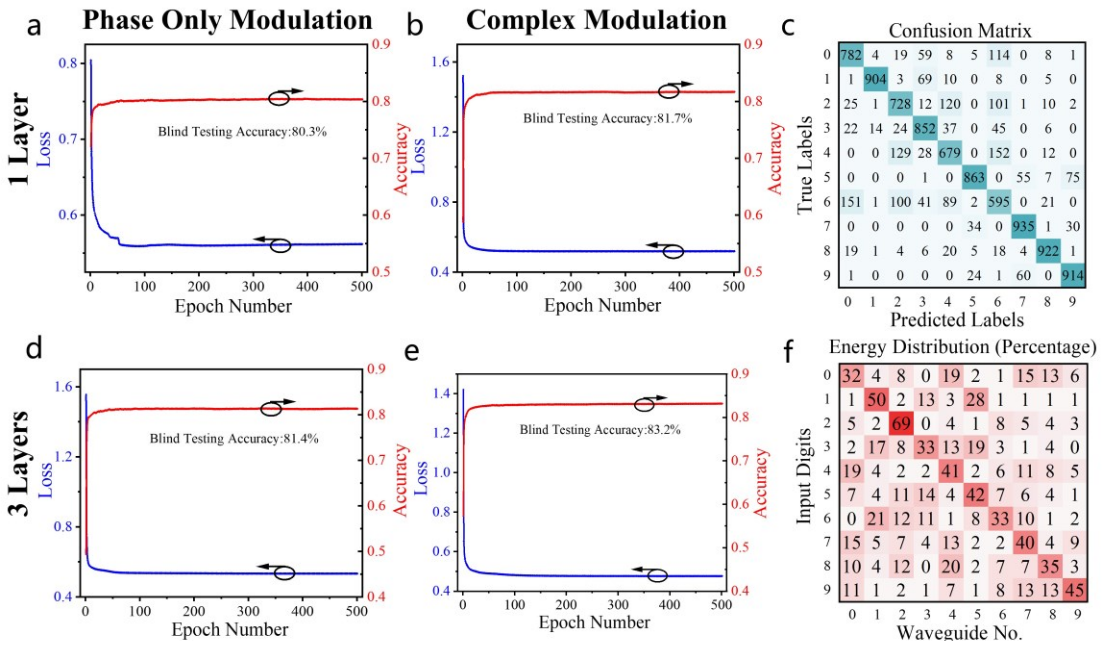

Fig. S13. *Fashion-MNIST* results achieved with the IDNN framework. a. b. d. e. Training convergence plots of phase-only as well as complex-valued modulation IDNNs (for 1 layer and 3 layers). The y-axis values in each plot report the Fashion MNIST classification accuracy and the loss values as a function of the epoch number for the testing datasets. **c. f.** Confusion matrix and energy distribution percentage for our numerical testing results for 10,000 different *Fashion-MNIST* dataset (~1000 for each image), achieving a classification accuracy of 85.2%.

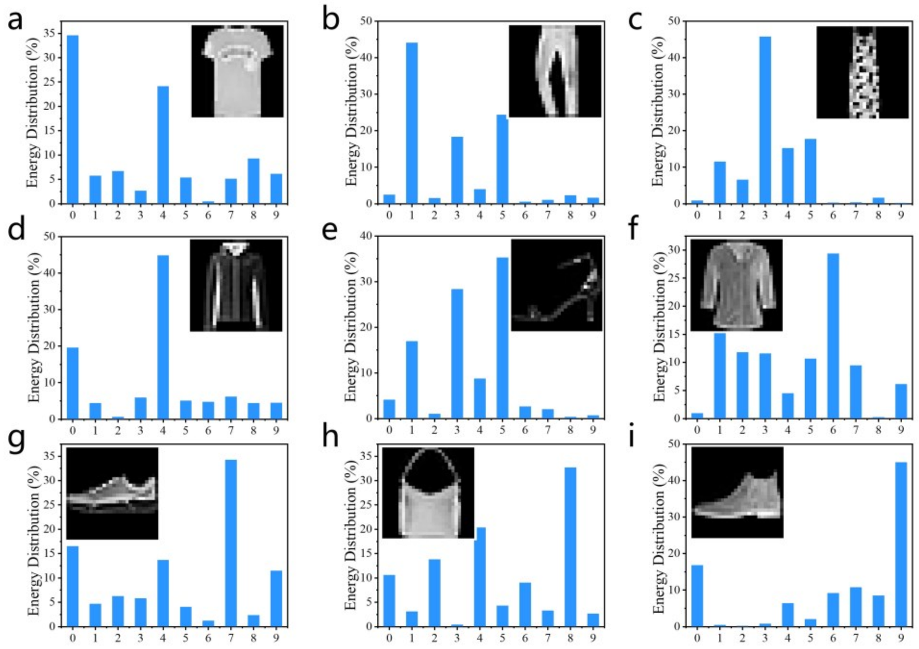

Fig. S14. Sample experimental results for fashion product classifier IDNN. Summary of some of the experimental results achieved with fashion product classification IDNN. The energy distribution percentage corresponding to each product at the output plane shows that IDNN has the maximum energy focused on the target detector region of each product (also see Fig. 5 of the main text).

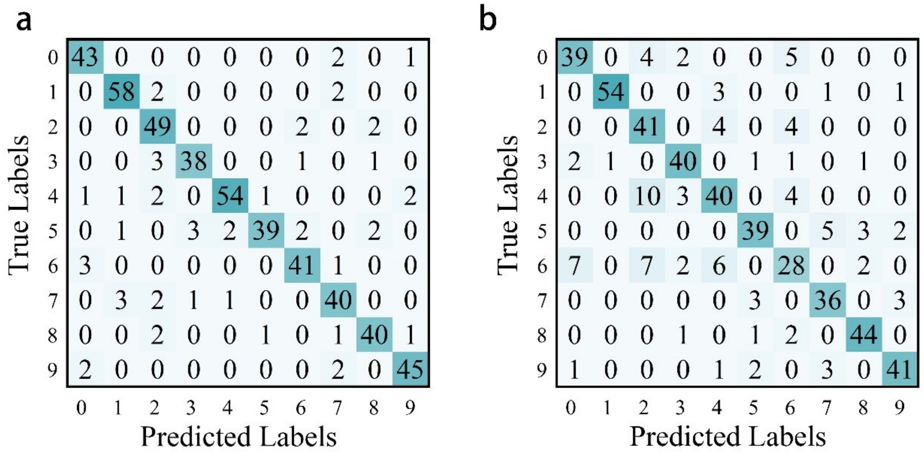

Fig. S15 a The confusion matrix for experimental results using 500 different samples with **a** phase encoding of *MNIST* dataset; and **b** amplitude encoding of *Fashion-MNIST* dataset.

Supplementary Note 10: Error sources

Four main sources of error affect the performance of an IDNN: (1) Alignment errors during the phase calibration process. The heaters used to modulate the phase have a theoretical resolution and the output intensity used to calibrate the phase also has certain fluctuations. These two parts will generate alignment errors in the phase calibration process. (2) Thermal crosstalk. For a thermal phase shifter, heat will be generated in the process of phase-shifting, which will result in thermal crosstalks between different MZI units. In our experiment, thermal isolation trenches and a temperature controller are used to reduce the residual thermal crosstalk. The isolation trenches are fabricated by deep etching the SiO_2 top cladding and Si substrate. A thermoelectric controller is placed underneath the chip to reduce the temperature fluctuation. (3) Splitting ratio error. Due to the imperfect 50/50 beam splitters in MZIs, the splitting ratio will cause a little detuning. In our experiment, this detuning is smaller than 3%, which can be ignored. (4) Diffraction errors in diffractive cells. The diffraction errors are composed of two parts: The light from the waveguide is Gaussian, resulting in a loss deviation between output waveguides; the output coupling loss of different waveguides is not uniform. The two kinds of errors can be compensated by introducing a calibration matrix.

Supplementary References

1. Shen, Yichen, et al. Deep learning with coherent nanophotonic circuits. Nat. Photonics 11, 441 (2017).

2. Ribeiro, A. et al. Demonstration of a 4×4-port universal linear circuit. Optica 3, 1348–1357 (2016).

3. Zhang, H. et al. An optical neural chip for implementing complex-valued neural network. Nat. Commun. 12, 1-11 (2021).

4. Caiwen, D. et al. Structured weight matrices-based hardware accelerators in deep neural networks: Fpgas and asics. Proceedings of the 2018 on Great Lakes Symposium on VLSI. (2018).

5. Goodman J. W. Introduction to Fourier optics[M]. Roberts and Company Publishers (2005).

6. Koichi, T. et al. Optical orthogonal frequency division multiplexing demultiplexer using slab star coupler-based optical discrete Fourier transform circuit. Opt. Lett. 36, 1140-1142 (2011).
